# 711 Regional Burn Review: Brooke or Parkland, neither reaches the 85% accuracy mark for burn resuscitation

**DOI:** 10.1093/jbcr/irac012.265

**Published:** 2022-03-23

**Authors:** Nava Azimzadeh, Rachel A Dahl, Colette Galet, Lucy Wibbenmeyer

**Affiliations:** University of Iowa, Coralville, Iowa; University of Iowa, Iowa City, Iowa; University of Iowa, Iowa City, Iowa; University of Iowa MD, FACS, Iowa City, Iowa

## Abstract

**Introduction:**

Much has been written about burn resuscitation and, despite preponderant studies, the topic remains controversial. Over-resuscitation can result in burn wound conversion and other complications. Our team switched from using modified Parkland (PF) to Brooke formula (BF) in January 2020. Secondary to difficult resuscitations, we sought to review our data to identify factors associated with over-resuscitation using either formula. We hypothesized that the Brooke formula under-predicted resuscitation volumes compared to Parkland, particularly in older patients or in more severe burn injuries.

**Methods:**

Medical records from patients who were admitted to the burn unit between 1/1/2019 and 8/29/2021 for a burn injury with %TBSA ≥15% were retrospectively reviewed. Exclusion criteria included age < 18 years, weight < 30 kg, patient not requiring resuscitation, or death within 24 h of admission. Demographics, injury information, resuscitation formula used, predicted 24-h resuscitation goal, the volume of resuscitation fluids, and time to reach maintenance were collected. Univariate analysis was performed to compare patients resuscitated using the BF vs. the PF. Multivariate analysis was performed to identify factors associated with over-resuscitation. Over resuscitation was defined as >2.5 mL/kg/%TBSA for BF and >5 mL/Kg/%TBSA for PF. P < 0.05 was considered significant.

**Results:**

Sixty-four patients were included; 27 were resuscitated using BF and 37 using PF. Neither age, sex, weight, BMI, or severity of burn injury were significantly different between the groups. Patients required a median of 3.59 mL/Kg/%TBSA for BF and 3.99 mL/kg/%TBSA for PF, p = 0.32. Over-resuscitation was more likely to occur when using BF compared to PF (59.3% vs. 32.4%, p = 0.043). Stepwise multivariate logistic regression showed that over-resuscitation was associated with longer time to reach maintenance (OR = 1.179 [1.042-1.333], p = 0.009) and arrival through ground transportation (OR = 10.523 [1.171-94.597], p = 0.036). Patients who arrived through ground transportation took longer to reach the burn center (3.98 h [2.65-5.15] vs. 2.57 h [1.93-3.2], p = 0.004) and presented with lower %TBSA (20.8 [18-27.9] vs. 31.3[21-55], p = 0.004).

**Conclusions:**

Time to reach definitive care was the only independent variable associated with over-resuscitation. While not significant, compared to PF, using BF tended to be associated with over-resuscitation. More studies are warranted to determine if BF continues to underperform and, if so, the populations in which it underperforms will be identified.

